# Epidemiological and clinical characteristics predictive of ICU mortality of patients with traumatic brain injury treated at a trauma referral hospital – a cohort study

**DOI:** 10.1186/s12883-023-03145-2

**Published:** 2023-03-08

**Authors:** Álvaro Réa-Neto, Elizeu Daniel da Silva Júnior, Gabriela Hassler, Valkiria Backes dos Santos, Rafaella Stradiotto Bernardelli, Amanda Christina Kozesinski-Nakatani, Marcelo José Martins-Junior, Fernanda Baeumle Reese, Mariana Bruinje Cosentino, Mirella Cristine Oliveira, Hélio Afonso Ghizoni Teive

**Affiliations:** 1 Center for Studies and Research in Intensive Care Medicine (CEPETI), Monte Castelo Street, 366, Curitiba, Paraná 82530-200 Brazil; 2 Internal Medicine Department, Hospital de Clínicas, Federal University of Paraná, General Carneiro Street, 181, Curitiba, Paraná 80060-900 Brazil; 3 Federal University of Paraná, General Carneiro Street, 181, Curitiba, Paraná 80060-900 Brazil; 4 School of Medicine and Life Sciences, Pontifical Catholic University of Paraná, Imaculada Conceição Street, 1155, Curitiba, Paraná 80215-901 Brazil; 5 Hospital Santa Casa de Curitiba., Praça Rui Barbosa, 694, Curitiba, Paraná 80010-030 Brazil; 6 Complexo Hospitalar do Trabalhador (CHT), República Argentina Street, 4406, Curitiba, Paraná 81050-000 Brazil; 7 Neurology Service, Internal Medicine Department, Hospital de Clínicas, Federal University of Paraná, General Carneiro Street, 181, Curitiba, Paraná 80060-900 Brazil

**Keywords:** Traumatic brain injury, Craniocerebral trauma, Intensive care unit, Trauma

## Abstract

**Background:**

Traumatic brain injury (TBI) has substantial physical, psychological, social and economic impacts, with high rates of morbidity and mortality. Considering its high incidence, the aim of this study was to identify epidemiological and clinical characteristics that predict mortality in patients hospitalized for TBI in intensive care units (ICUs).

**Methods:**

A retrospective cohort study was carried out with patients over 18 years old with TBI admitted to an ICU of a Brazilian trauma referral hospital between January 2012 and August 2019. TBI was compared with other traumas in terms of clinical characteristics of ICU admission and outcome. Univariate and multivariate analyses were used to estimate the odds ratio for mortality.

**Results:**

Of the 4816 patients included, 1114 had TBI, with a predominance of males (85.1%). Compared with patients with other traumas, patients with TBI had a lower mean age (45.3 ± 19.1 versus 57.1 ± 24.1 years, p < 0.001), higher median APACHE II (19 versus 15, p < 0.001) and SOFA (6 versus 3, p < 0.001) scores, lower median Glasgow Coma Scale (GCS) score (10 versus 15, p < 0.001), higher median length of stay (7 days versus 4 days, p < 0.001) and higher mortality (27.6% versus 13.3%, p < 0.001). In the multivariate analysis, the predictors of mortality were older age (OR: 1.008 [1.002–1.015], p = 0.016), higher APACHE II score (OR: 1.180 [1.155–1.204], p < 0.001), lower GCS score for the first 24 h (OR: 0.730 [0.700–0.760], p < 0.001), greater number of brain injuries and presence of associated chest trauma (OR: 1.727 [1.192–2.501], p < 0.001).

**Conclusion:**

Patients admitted to the ICU for TBI were younger and had worse prognostic scores, longer hospital stays and higher mortality than those admitted to the ICU for other traumas. The independent predictors of mortality were older age, high APACHE II score, low GCS score, number of brain injuries and association with chest trauma.

## Introduction

Among traumatic injuries, traumatic brain injury (TBI) is associated with increased morbidity and mortality among adults worldwide, leading often disabling to physical and psychological consequences [1–3]. According to data from the Global Burden of Disease [4], the overall incidence of TBI in 2016 was 369 per 100,000 inhabitants, with a 4% increase in the incidence rate between 1990 and 2016. In comparison, in Brazil, the incidence of TBI in 2016 was similar to the global incidence, with 383 per 100,000 inhabitants, with a percentage increase of 5.6 [4]. Furthermore, between 2008 and 2012, TBI accounted for 9,700 hospital deaths per year, overwhelming the public health system [5].

In addition to the high incidence, a European study revealed that almost half of hospitalized patients with TBI require admission to an intensive care unit duo to the risks of secondary brain injuries and complications [6]. These patients evolve to death on average in 15% of cases or require prolonged periods of hospitalization [7].

Detailed and contextualized epidemiological and clinical information on individuals with TBI admitted to the ICU are important for understanding the risks associated with morbidity and mortality. In addition, such information can contribute to the development of therapeutic and preventive measures that can be applied during hospitalization.

Despite the consequences of TBI on public health, in Brazil, there are still few studies detailing clinical and epidemiological characteristics that predict outcomes for these patients in the ICUs [1, 8–10]. Thus, the aim of this study was to identify epidemiological and clinical characteristics predictive of ICU mortality among patients admitted for TBI in a trauma referral hospital.

## Materials and methods

This retrospective cohort study consecutively included patients with TBI over 18 years of age treated in the ICU between January 1, 2012, and August 31, 2019, in a trauma referral hospital in the city of Curitiba/PR, Brazil. The Complexo Hospitalar do Trabalhador is a level 1 trauma center according to the American Trauma Society classification, with a tertiary care facility available to the public health system and capable of providing comprehensive care for all aspects of injuries [11]. Patients are referred by the public health system center and the hospital follows the recommendations of the Brain Trauma Foundation [12]. All patients with an altered Glasgow Coma Scale or any acute abnormality on CT and who had an indication of full support at hospital admission were admitted to the ICU.

Patients who were hospitalized for late sequelae of TBI and those for whom there ware no data on age, sex, and type of TBI on the CT or ICU outcome recorded in the electronic medical record and in the daily medical follow-up sheets at the bedside were excluded.

The study was approved by the local ethics committee of the Instituto de Neurologia de Curitiba under protocol number 5.663.561 (Project title: Factors associated with mortality and hospital time in patients with traumatic brain (TBI) in intensive care units (ICU) in a trauma reference hospital in the city of Curitiba/PR, Brazil between 2012 and 2019; CAAE:61409122.9.0000.5227) and the need for informed consent was waived given the noninterventional study design and data collection was performed only in clinical records, without contact with the participants. All research procedures were conducted in accordance with the ethical standards of the institutional committee on human experimentation and with the Helsinki Declaration of 1975 and Resolution 466/12 of the National Health Council (Conselho Nacional de Saúde - CNS). The STROBE guidelines were used to ensure the reporting of this study.

Using electronic medical records and daily bedside medical tracking forms, data were collected on the sex and age of the patient and on the mechanism of trauma (e.g., gunshot wound (GSW); stab wound (SW); physical aggression; being run over; bicycle accident; collisions; motorcycle accident; and falling from the same level (SLF) and/or falling from a height (OLF). In addition, information was collected on the presence of polytrauma and/or skull trauma, and injuries identified on cranial CT were recorded as follows: subarachnoid haemorrhage (SAH), subdural haematoma (SDH), epidural haematoma (EDH), diffuse axonal injury (DAI), intraparenchymal haemorrhage (IPH), cerebral contusion, skull depression, skull fracture, intraventricular haemorrhage, ischaemia, cerebral oedema and/or pneumocephalus.

Data were also collected on the type and number of TBI-associated injuries, which may present as injuries to chest, abdomen, pelvis, spine, limbs and/or face and neck. The types of interventions ware recorded: conservative treatment, intracranial pressure monitoring (ICPm), craniectomy, hematoma drainage, external ventricular shunt(EVD) and correction of depression and/or fracture correction. Last, the following data for ICU admission were collected: SOFA (Sequential Organ Failure Assessment) score, APACHE II (Acute Physiology and Chronic Health Evaluation) score and Glasgow Coma Scale (GCS) scores and outcome (length of hospitalization, discharge or death, reason for death and GCS score at discharge).

### Statistical analysis

The results for categorical variables are presented as absolute and relative frequencies, and the results for quantitative variables are presented as the mean and standard deviation, median and minimum and maximum values.

For quantitative variables, comparisons between 2 groups were performed using Student’s t test or the nonparametric Mann‒Whitney test for data with a nonnormal distribution. The association between 2 categorical variables was performed using the chi-square test, and when both were dichotomous, Fisher’s exact test was applied.

Simple and multiple binary logistic regression models of the following factors alone and/or adjusted were used to estimate odds ratios and 95% confidence intervals for mortality: age, trauma mechanisms, APACHE II score, GCS score, SOFA score in the first 24 h, associated injuries, number of injuries on cranial CT and presence of each brain injury on CT, compared with the absence of any injury. The significance of each of the variables in the models was evaluated using the Wald test.

The level of statistical significance was set at 5%, and the data were analysed using the statistical analysis software IBM SPSS, version 28.0 (SPSS Inc., Chicago, IL, USA). Imputation of missing data was not performed.

## Results

**Fig. 1:**
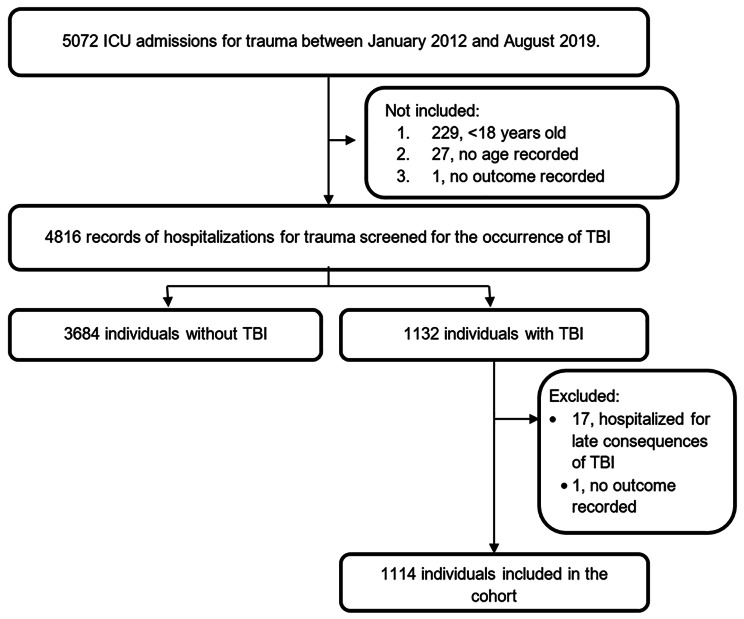
Process of selection of the study sample Abbreviations: ICU, intensive care unit; TBI, traumatic brain injury

Between January 2012 and August 2019, 5,072 trauma victims were admitted to the ICU. Of these, 4816 patients older than 18 years at the time of ICU admission were screened for TBI, of which 23.5% were diagnosed with a TBI (n = 1132). We excluded 17 patients hospitalized for late sequelae of TBI and 1 who was transferred to the ICU of another hospital, making it impossible to determine the outcome. Thus, 1,114 patients made up the cohort of this study (Fig. 1).

Comparing the profile of the 1,114 individuals with TBI with that of the 3,684 individuals with other traumas (Table 1) indicated that those admitted to the ICU for TBI were significantly younger and had worse prognostic scores on admission (APACHE II, SOFA and GCS scores); they remained hospitalized longer, and there was a higher proportion of deaths in this group.

**Table 1:** Characteristics of patients admitted for trauma and a comparison between those with and without TBI

Variables	Total (n = 4798)	Without TBI (n = 3684)	With TBI (n = 1114)	P -value
**Male sex**, n (%)	3180 (66.3)	2232 (60.6)	948 (85.1)	< 0.001^b^
**Age (years)**, mean ± SD	54.4 ± 23.5	57.1 ± 24.1	45.3 ± 19.1	< 0.001^c^
**Apache II score**, mean; med. (min-max)	17.2; 16 (0–59)	16.4; 15 (0–59)	20; 19 (0–49)	< 0.001^d^
**Worst Glasgow Coma Scale score in the 1st 24 h**, mean; med. (min-max)	12; 14 (3–15)	13.1; 15 (3–15)	9; 10 (3–15)	< 0.001^d^
**SOFA score in the 1st 24 h**^**a**^, mean; med. (min -max)	4.9; 4 (0–21)	4.4; 3 (0–21)	6.5; 6 (0–18)	< 0.001^d^
**Length of ICU stay**, mean; med. (min-max)	9; 5 (0-141)	8.5; 4 (0-141)	10.8; 7 (1–90)	< 0.001^d^
**ICU outcome**, n (%)				< 0.001^d^
Discharge	4001 (83.4)	3194 (86.7)	807 (72.4)	
Death	797 (16.6)	490 (13.3)	307 (27.6)	

^**a**^ Valid n of 4221 for the total, 3247 for the group without TBI and 974 for the group with TBI

^**b**^ Significant, Fisher’s exact test

^**c**^ Significant, Student’s t test for independent samples

^**d**^ Significant, nonparametric Mann‒Whitney test

Abbreviations: ICU, intensive care unit; TBI, traumatic brain injury; APACHE, Acute Physiology and Chronic Health Evaluation; SOFA, Sequential organ failure assessment

p < 0.05 indicates statistical significance

**Fig. 2:**
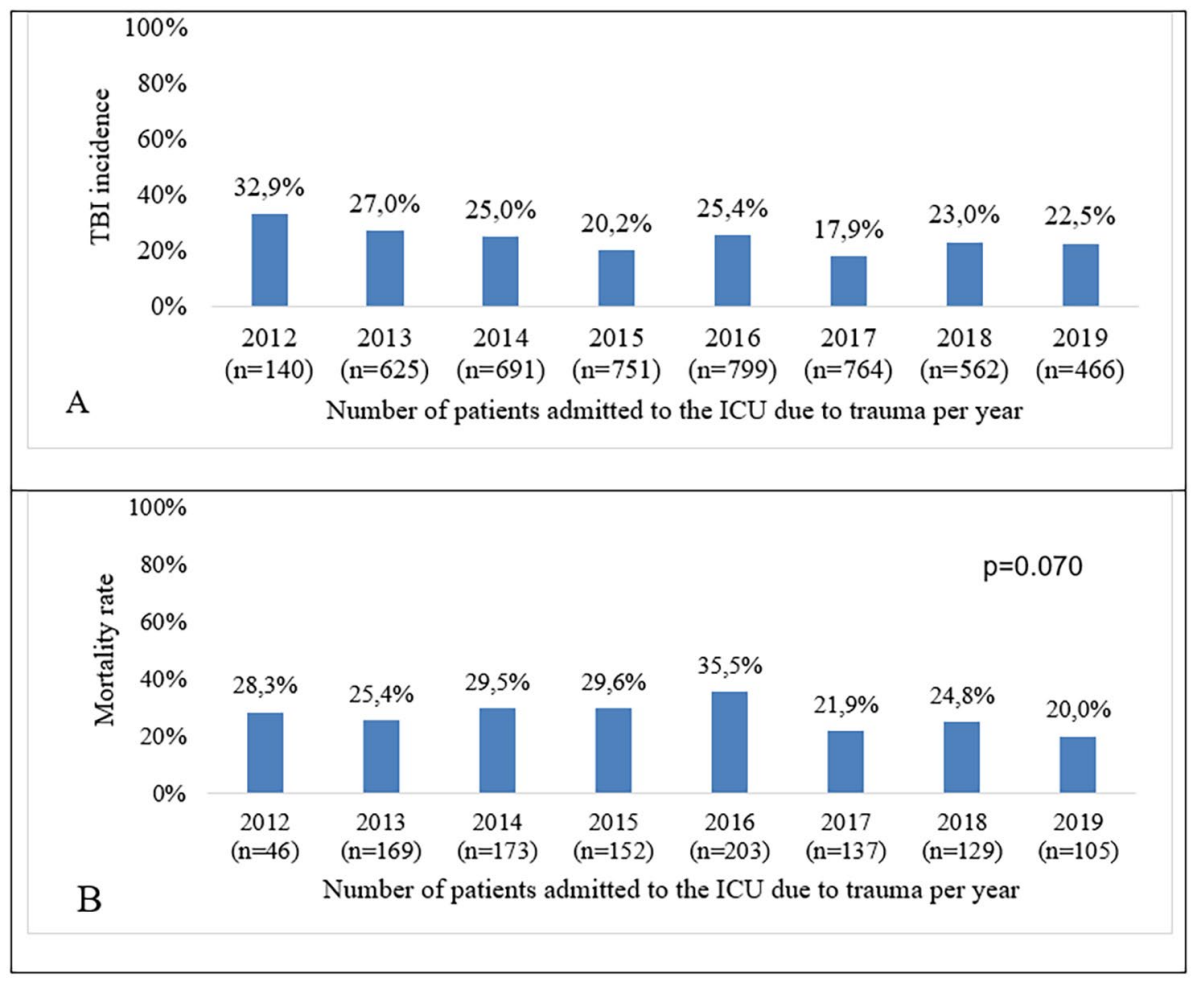
Annual incidence and mortality rate for TBI in ICU. **A**: Annual incidence of ICU admissions due to TBI among traumatic reasons. **B**: Mortality rate for TBI victims admitted to the ICU per year Abbreviations: ICU, intensive care unit; TBI, traumatic brain injury

The 1114 individuals with TBI accounted for 23.2% of trauma patients admitted to the ICU from 2012 to August 2019. The annual incidence of ICU admission by TBI ranged from 32.9 to 17.9%, with the lowest incidence recorded in 2017 (Fig. 2A). The overall mortality rate for the individuals with TBI was 27.6%, with no significant difference between the rates in the years evaluated (Fig. 2B).

Of the 1,114 patients admitted to the ICU due to TBI, 27.6% died. Among them, the mean age was 47.6 ± 19.7 years, higher than those who progressed to discharge. Among those with TBI, there was a predominance of males, with no difference in the proportion of deaths between the sexes (Table 2).

**Table 2:** Description of the characteristics of individuals with TBI and their association with ICU outcome

VARIABLES	Total (n = 1114)	Discharge	Death	p value
ICU ADMISSION				
**Age (years)**, mean ± SD	45.3 ± 19.1	44.5 ± 18.9	47.6 ± 19.7	0.016^b^
**Male**, n (%)*	948 (85.1)	689 (72.7)	259 (27.3)	0.707^c^
**Trauma mechanism**, n (%) *				< 0.001^a^
GSW/SW	77 (7.4)	47 (56.6)	36 (43.4)	
Physical Aggression	142 (12.7)	113 (79.6)	29 (20.4)	
Run over	158 (14.2)	104 (65.8)	54 (34.2)	
Cyclist	50 (4.5)	29 (58)	21 (42)	
Collision	93 (8.3)	73 (78.5)	20 (21.5)	
Motorbike	203 (18.2)	159 (78.3)	44 (21.7)	
SLF	238 (21.4)	172 (72.3)	66 (27.7)	
OLF	147 (13.2)	110 (74.8)	37 (25.2)	
**Passage to the operating room before ICU addition**, n(%)				0,033^c^
Yes	789 (70.8)	557 (70.6)	232 (29.4)	
No	325 (29.2)	250 (76.9)	75 (23.1)	
**Apache II score**, mean; med. (min-max)	20; 19 (0–49)	16.5; 15 (0–41)	29.1; 30 (6–49)	0.000^d^
**Worst Glasgow Coma Scale (GCS) in the first 24 h**, mean; med. (min-max)	9; 10 (3–15)	10.5; 12 (3–15)	5.3; 3 (3–15)	0.000^d^
**Classification of Severity of TBI by the worst GCS in the first 24 h**, n(%)				< 0,001^a^
Severe (GCS 3 to 8)	519 (46.6)	264 (50.9)	255 (49.1)	
Moderate (GCS 9 to 12)	205 (18.4)	176 (85.9)	29 (14.1)	
Mild (GCS 13 to 15)	390 (35.0)	367 (94.1)	23 (5.9)	
**SOFA score in the first 24 h**, mean; med. (min-max) ^#^	6.5; 6 (0–18)	5.4; 5 (0–16)	9.5; 9 (2–18)	0.000^d^
Number of injuries on cranial CT, n (%) *				< 0.001^a^
None	43 (3.9)	39 (90.7)	4 (9.3)	
One	736 (66.1)	552 (75)	184 (25)	
Two	250 (22.4)	176 (70.4)	74 (29.6)	
Three	85 (7.6)	40 (47.1)	45 (52.9)	
**Injuries evidenced on CT**, n (%) *				
Cerebral contusion	167 (15.0)	122 (73.1)	45 (26.9)	0.015^e^
DAI	47 (4.2)	40 (85.1)	7 (14.9)	0.527^e^
Fracture and/or depression	99 (8.9)	70 (70.7)	29 (29.3)	0.009^e^
SAH	379 (34.0)	244 (64.4)	135 (35.6)	< 0.001^e^
SDH	401 (36.0)	268 (66.8)	133 (33.2)	< 0.001^e^
EDH	151 (13.6)	127 (84.1)	24 (15.9)	0.335^e^
IPH	149 (13.4)	112 (75.2)	37 (24.8)	0.034^e^
Haemoventriculum	19 (1.7)	8 (42.1)	11 (57.9)	< 0.001^e^
Cerebral edema	55 (4.9)	17 (30.9)	38 (69.1)	< 0.001^e^
Ischaemia	8 (0.7)	2 (25)	6 (75)	< 0.001^e^
Pneumocephalus	13 (1.2)	11 (84.6)	2 (15.4)	0.615^e^
**Presence of polytrauma**, n (%) *	318 (28.5)	230 (72.3)	88 (27.7)	1^c^
**Number of injury follow-ups in addition to TBI**, n (%) *				0.260^a^
None	796 (71.5)	577 (72.5)	219 (27.5)	
One	190 (17.1)	142 (74.7)	48 (25.3)	
Two	94 (8.4)	61 (64.9)	33 (35.1)	
Three or more	34 (3.1)	27 (79.4)	7 (20.6)	
**Injury follow-up in addition to TBI**, n (%) *				
Thorax	140 (44)	87 (62.1)	53 (37.9)	0,015^f^
Abdomen	56 (17.6)	37 (66.1)	19 (33.9)	0,355^f^
Pelvis	14 (4.4)	11 (78.6)	3 (21.4)	0,768^f^
SCI	59 (18.6)	44 (74.6)	15 (25.4)	0,880^f^
Limbs	129 (40.6)	93 (72.1)	36 (27.9)	0,916^f^
Face and/or neck (except spine)	84 (26.4)	75 (89.3)	9 (10.7)	< 0,001^f^
**TREATMENT OF TBI**				
**Approach to TBI treatment**, n (%) *				0,138^c^
Conservative	494 (44.3)	369 (74.7)	125 (25.3)	
Surgical	620 (55.7)	438 (70.6)	182 (29.4)	
**Procedures performed to treat TBI**, n (%) *				
ICPm	418 (67.4)	272 (65.1)	146 (34.9)	0,002^ g^
Craniectomy	158 (25.5)	86 (54.4)	72 (45.6)	< 0.001^ g^
Haematoma drainage	140 (22.6)	122 (87.1)	18 (12.9)	0.002^ g^
EVD	94 (15.5)	71 (75.5)	23 (24.5)	1^ g^
Correction of fracture and/or depression	8 (1.3)	8 (100)	0 (0)	0.210^ g^
**ICU OUTCOME**				
**Length of stay**, mean; med. (min-max)	10.8; 7 (1–90)	12; 8 (1–90)	7.4; 5 (1–74)	< 0.001^d^
**Level of LST**, n (%) ^§^				< 0.001^a^
LST-A	803 (74.1)	742 (92.4)	61 (7.6)	
LST-B	74 (6.8)	24 (32.4)	50 (67.6)	
LST-C	77 (7.1)	14 (18.2)	63 (81.8)	
LST-D	17 (1.6)	1 (5.6)	17 (94.4)	
LST-E	113 (10.4)	0 (0)	113 (100)	

**Abbreviations:** TBI, traumatic brain injury; ICU, intensive care unit; OR, odds ratio; GSW, Gunshot wound; SW, Stab wound; SLF, Same-level fall; OLF, Fall from height; SCI, spinal cord injure; APACHE, Acute Physiology and Chronic Health Evaluation; CT, computed tomography; DAI, Diffuse axonal injury; SAH, Subarachnoid haemorrhage; SDH, subdural haematoma; EDH, epidural haematoma; IPH, Intraparenchymal haemorrhage; ICPm, intra cranial pressure monitoring; EVD, External ventricular derivation; LST: life-sustaining treatments; LST-A: All necessary, possible, and available LST measures to save life and restore health, including cardiopulmonary resuscitation if cardiopulmonary arrest; LST-B: All necessary, possible, and available LST measures to save life and restore health, but no cardiopulmonary resuscitation if cardiopulmonary arrest; LST-C: Maintenance of LST measures already in place, withholding new ones apart from those aimed at comforting the patient and his or her family; LST-D: Removal of LST measures when considered futile in the face of a terminal condition and beginning of palliative care; LSTT-E: All necessary, possible and available LST measures for patients with brain death until organ donation

^*^ For the variables in the column “Total”, the percentages calculated are presented considering the total number of cases in the column, while in the columns “Discharge” and “Death”, the percentages calculated considering the total number of cases in the row are presented

^**#**^ 140 missing data points in the total sample (88 among discharges and 52 among deaths)

^**§ **^ 30 missing data points in the total sample (26 among discharges and 4 among deaths)

^**a**^ Significant, chi-square test

^**b**^ Significant, Student’s t test for independent samples

^**c**^ Significant, Fisher’s exact test

^**d**^ Significant, nonparametric Mann‒Whitney test

^**e**^ Significant, Fisher’s exact test, compared with the 43 patients who did not present any injury on cranial CT.

^**f**^ Significant, Fisher’s exact test, compared with the 796 patients who had no record of injuries

^**g**^ Significant, Fisher’s exact test, compared with the 494 patients who underwent conservative treatment

The most common trauma mechanisms were falls from the same level followed by motorcycle accidents, corresponding to 39.6% of all TBIs. However, the TBI mechanism with the highest mortality rate was GSW/SW, followed by bicycle accidents and being run over. There was a significant difference in the proportion of deaths between the trauma mechanisms (Table 2).

More than 60% of patients had only one injury on cranial CT, and the increase in the number of associated injuries was directly related to mortality. The most frequent injuries evidenced on tomography were SDH and SAH. Patients with ischaemia, cerebral oedema and intraventricular haemorrhage had the highest mortality rates. In addition, compared toother injuries, a significantly higher proportion of patients with brain contusions, skull fractures, SAH, SDH, IPH, intraventricular haemorrhage, cerebral oedema and ischaemia died (p < 0.05) (Table 2).

The majority, 46.6%, were characterized as severe TBI (GCS 3 to 8), 18.4% as moderate (GCS 9 to 12) and 35% as mild (GCS 13 to 15). The presence of severe TBI was significantly associated with mortality. Furthermore, patients who died had significantly lower GCS scores and higher APACHE II and SOFA scores in the first 24 h in ICU. The presence of trauma in other areas, except chest trauma was not significantly related to death. (Table 2).

Most TBIs were treated surgically, with no difference in the mortality rate between surgical and conservative treatment. Among the surgical procedures, the most frequent was ICPm, and the approach associated with higher mortality was decompressive craniectomy. The median length of stay in the ICU was 7 days, which was significantly longer among those who died. At the time of ICU outcome, the majority (74.1%) of the patients were classified as life-sustaining treatments (LST) level A i.e., all necessary, possible, and available LST measures to save life and restore health, including cardiopulmonary resuscitation if cardiopulmonary arrest. (Table 2).

Of the 807 survivors, 781 (96.8%) had the Glasgow Coma Scale recorded at ICU discharge. The mean score was 13.2, and the median was 14, ranging from 3 to 15. Among the 307 patients who died, the direct cause of death was noted in the discharge summary of 262 (85.3%) patients. Brain death was the most frequent, accounting for 42.7% of deaths, followed by infection (40.8%), hemorrhagic shock (4.6%), unidentified cause (3.4%), pulmonary thromboembolism (PTE) (1.9%), sepsis plus acute respiratory distress syndrome (ARDS) (1.5%), ARDS alone (1.1%), acute myocardial infarction (AMI) (1.1%), cardiac arrest (CA) duo toventricular fibrillation (VF) (1.1%), and others (1.5%).

Variables that were significantly different between discharge and death were analysed as prognostic factors for mortality using univariate logistic regression analysis (Table 3). Older age, higher APACHE II and SOFA scores and lower GCS scores at admission were associated with higher mortality. Regarding TBI mechanisms, less lethal trauma (physical aggression), GSW/SW, being run over and bicycle accidents were associated with a higher risk of mortality (Table 3).

**Table 3:** Univariate model of prognostic factors for mortality among TBI patients admitted to the ICU.

Prognostic factors	N of the model	OR (95% CI) for mortality^a^	p value^b^
**Age (years)**	1114	1.008 (1.002–1.015)	0.016
**Trauma mechanism**	1114		
Physical Aggression		Ref	
GSW/SW		2.985 (1.645–5.415)	0.000
Run over		2.023 (1.198–3.417)	0.008
Cyclist		2.822 (1.409–5.649)	0.003
Collision		1.068 (0.562–2.027)	0.842
Motorbike		1.078 (0.636–1.827)	0.779
SLF		1.495 (0.91–2.458)	0.113
OLF		1.311 (0.754–2.277)	0.337
**Apache II score**	1114	1.180 (1.155–1.204)	< 0.001
**Worst Glasgow Coma Scale score in the first 24 h**	1114	0.730 (0.700–0.760)	< 0.001
**Classification of Severity of TBI by the worst GCS in the first 24 h**	1114		
Severe (GCS 3 to 8)		Ref	
Moderate (GCS 9 to 12)		2.629 (1.478–4.677)	0,001
Mild (GCS 13 to 15)		15,413 (9,778–24,295)	< 0,001
**SOFA score in the first 24 h**	974	1.400 (1.330–1.473)	< 0.001
**Number of injuries on cranial CT**	1114		
None		Ref	
One		3.250 (1.146–9.218)	0.027
Two		4.099 (1.414–11.883)	0.009
Three		10.969 (3.602–33.406)	< 0.001
**The presence of each of brain injury evidenced on cranial CT compared to having no injuries on CT**			
**Cerebral contusion** (ref: 43 patients without injury)	210	3.596 (1.216–10.636)	0.021
**DAI** (ref: 43 patients without injury)	90	1.706 (0.463–6294)	0.422
**Fracture and/or depression** (ref: 43 patients without injury)	142	4.039 (1.323–12.335)	0.014
**SAH** (ref: 43 patients without injury)	422	5.394 (1.887–15.420)	0.002
**SDH** (ref: 43 patients without injury)	444	4.839 (1.694–13.824)	0.003
**EDH** (ref: 43 patients without injury)	194	1.842 (0.603–5.634)	0.284
**IPH** (ref: 43 patients without injury)	192	3.221 (1.079–9.619)	0.036
**Haemoventriculum** (ref: 43 patients without injury)	62	13.406 (3.393–5 2.977)	< 0.0001
**Cerebral edema** (ref: 43 patients without injury)	98	21.794 (6.715–70.732)	< 0.001
**Ischaemia** (ref: 43 patients without injury)	51	29.250 (4.364–196.069)	< 0.001
**Pneumocephalus** (ref: 43 patients without injury)	56	1.773 (2.86–10.990)	0.539
**Presence of trauma in another segment in addition to the skull**	1114	1.008 (0.754–1.348)	0.957
**Number of injured segments in addition to TBI**	1114		
None		Ref	
One		0.891 (0.620–1.280)	0.531
Two		1.425 (0.908–2.238)	0.124
Three or more		0.683 (0.293–1.591)	0.377
**Injury follow-up in addition to TBI**			
**Associated thorax injury** (ref: absence of thoracic injury)	1114	1.727 (1.192–2.501)	0.004
**Associated abdominal injury** (ref: absence of abdominal injury)	1114	1.373 (0.777–2.427)	0.275
**Associated pelvis injury** (ref: absence of pelvis injury)	1114	0.714 (0.198–2.577)	0.607
**Associated MRT** (ref: absence of MRT)	1114	0.891 (0.488–1.625)	0.706
**Associated limb injury** (ref: absence of limb injury)	1114	1.020 (0.677–1.536)	0.925
**Associated face and/or neck injury (except spine)** (ref: absence of face/neck injury)	1114	0.295 (0.146–0.596)	< 0.001

**Abbreviations:** TBI, traumatic brain injury; ICU, intensive care unit; OR, odds ratio; GSW, Gunshot wound; SW, Stab wound; SLF, Same-level fall; OLF, Fall from height; APACHE, Acute Physiology and Chronic Health Evaluation; CT, computed tomography; DAI, Diffuse axonal injury; SAH, Subarachnoid haemorrhage; SDH, subdural haematoma; EDH, epidural haematoma; IPH, Intraparenchymal haemorrhage

^**a**^ Odds ratio (OR) and 95% confidence interval of the OR (95% CI) of the univariate binary logistic regression model for mortality

^**b**^ Significant, Wald test of the logistic regression model

The greater the number of injuries on cranial CT, the greater the risk of mortality when compared with the absence of brain lesion; patients with 3 injuries on cranial CT were 10 times more likely to die. The presentation of ischaemia, intraventricular haemorrhage, cerebral oedema, cerebral contusions, skull fractures, SAH, SDH and/or IPH increased the odds of death of patients with TBI. Cerebral ischaemia and oedema increased the odds by 29 and 21 times, respectively (Table 3). Polytrauma was not associated with a worse outcome; however, the presence of associated chest injury increased the odds of death (Table 3).

Finally, older age, a higher APACHE II score, a lower GCS score in the first 24 h, a greater number of brain injuries identified on CT, and the presence of associated chest trauma remained risk factors for mortality in patients with TBI after adjusting for each in a multiple regression model (Table 4).

**Table 4:** Multiple logistic regression of prognostic factors for mortality among patients with TBI in ICU

Prognostic factors	N of the model	OR (95% CI) for mortality^a^	P value^b^
**Age (years)**	1114	1.008 (1.002–1.015)	0.016
**Apache II**		1.180 (1.155–1.204)	< 0.001
**Worst Glasgow Coma Scale score in the first 24 h**		0.730 (0.700–0.760)	< 0.001
**Number of injuries on cranial CT**			
None		Ref	
One		3.250 (1.146–9.218)	0.027
Two		4.099 (1.414–11.883)	0.009
Three		10.969 (3.602–33.406)	< 0.001
**Associated thorax injury** (ref: absence of thoracic injury)		1.727 (1.192–2.501)	< 0.001

^**a**^ Odds ratio (OR) and 95% confidence interval of the OR (95% CI) of the multiple binary logistic regression model for mortality

^**b**^ Significant, Wald test of the logistic regression model

Abbreviations: TBI, traumatic brain injury; ICU, intensive care unit; OR, odds ratio; APACHE, Acute Physiology and Chronic Health Evaluation; CT, computed tomography

## Discussion

Compared with other severe traumas, TBI is the most prevalent and, among traumas, has the highest morbidity and mortality [13, 14]. In trauma, injuries to the central nervous system are the main causes of death, followed by hemorrhagic shock and sepsis [14–16]. Individuals with TBI are younger and mostly men [17–19] and have worse prognostic score values at admission and longer ICU stays. As observed in Europe, patients with neurological injuries admitted to the ICU had higher SOFA scores, lower GCS scores on admission, longer ICU stays and higher mortality rates [20].

In this study, the mortality rate for patients with TBI was 27.6%. Older age, a higher APACHE II score, a lower Glasgow score in the first 24 h, a higher number of brain injuries identified on CT, and the presence of associated chest trauma were independent predictors of mortality.

The findings corroborate previous studies that identified older age and lower GCS scores as predictors of mortality in patients with severe TBI [7, 17, 21–25]. A population based study conducted in Rwanda, Africa, showed that age over 50 years and GCS score lower than 13 were significantly associated with death [23]. In previous studies, younger patients had higher GCS scores at ICU admission and discharge [26], and mortality increased with increasing age [14, 27]. One explanation for the increased mortality in patients with advanced age is the increased use of anticoagulant and antiplatelet drugs, leading to greater bleeding complications in patients with severe trauma [13].

Falls from the same level and traffic accidents were the main trauma mechanisms identified, similar to those reported in European studies [18, 28]. Falls from the same level are more common in the elderly population, while traffic accidents are more common in the young population [17, 18, 29]. The higher incidence of falls in the elderly population can be explained by their increased in life expectancy accompanied by the increase in comorbidities [13]. For TBIs caused by traffic accidents, the most common mechanism among our cohort was motorcycle accidents, a finding that is different from observations in Europe, where bicycle accidents were more frequent [28–30].

Subdural and subarachnoid haemorrhages were the most frequent findings on CT of the head of patients with TBI, a finding consistent with the results of a European study [6]. The increase in the occurrence of intracranial haemorrhages is associated with olderage and a decrease in GCS score [31]. The presence of SAH due to trauma is often accompanied by other intracranial haemorrhages [32]. When SAH is concomitant with 2 other intracranial haemorrhage, there is a 9-fold increase in mortality, and when associated with SDH, there is a 16-fold increase in the chance of death [25, 32]. Thus, a greater number of brain injuries, especially haemorrhagic ones, is associated with a greater chance of death.

The association of thoracic trauma with TBI significantly increased the mortality of patient in our study. These findings are corroborated by other studies, also demonstrating higher mortality among patients with pulmonary contusions [33], and that the presence of TBI and concomitant chest trauma significantly increased mortality and prolonged ICU stay and duration of mechanical ventilation [34].

Higher APACHE II scores are a risk factor for mortality in patients admitted to ICUs [35, 36]. In the present study, individuals with TBI admitted to the ICU had a mean APACHE score of 20 points, and among the patients who died, the mean score was 29 points. These data are very similar to those reported in a study conducted in Turkey [37], in which the mean APACHE II score was 30 points for patients with TBI who died. This scale has shown good performance in terms of discrimination, calibration, accuracy and satisfactory results in the prediction of ICU mortality in patients with TBI [35, 38].

In the univariate analysis, the SOFA score was significantly related to mortality, with higher scores for patients who died [38]. Zygun D et al. [39] demonstrated that the increased mortality of patients with TBI is related to higher scores on the cardiovascular component of the SOFA.

Regarding therapeutic management, no differences were observed in mortality between conservative or surgical intervention. However, in 2020, Gao et al. [21] showed that decompressive craniotomy and craniectomy reduced mortality in patients with severe TBI and no pupillary light reflex.

At the time of ICU outcome, the majority (74.1%) of the patients were classified as life-sustaining treatments (LST) level A. In our institutional protocol, in view of the brain trauma foundation protocols and focus on rehabilitation, it is recommended to wait 6 months for support limitation. These data are in agreement with the results of other authors who suggest caution in considering the withdrawal of care in patients with TBI duo to the delay in the recovery of the level of consciousness [40].

Due to the retrospective nature of this study, not all data were available, such as: Injury Severity Score (ISS), regional AIS (Abbreviated injury score), Marshal Score, morbidity, incidence of ARDS and pneumonia. Since data were collected exclusively from electronic medical records, information on prehospital care, pupil assessment, functional outcome, and haemostasis disorders was not available. Data collection was performed only at the time of hospitalization, it was not possible to control confounding factors regarding the evolution of each patient during hospitalization. However, the findings of our study, derived from the analysis of a large TBI patients in a trauma referral hospital, allow a broad understanding of the epidemiological profile of TBI patients.

In conclusion, individuals with TBI have higher chances of mortality and longer hospital stay, are younger, have higher APACHE II and SOFA scores and have lower GCS scores on ICU admission. Significantly associated predictors of ICU mortality include older age, higher APACHE II score, lower GCS score in the first 24 h, higher number of brain injuries, and concomitant chest trauma. TBI results in extensive consequences for patients’ quality of life and represents a substantial burden on health services. Therefore, understanding the risk factors and predictors of mortality is necessary for the development of more effective preventive and therapeutic measures and for better planning of the management of patients with TBI, in order to reduce morbidity and mortality.

## Data Availability

The dataset supporting the conclusions of this article is available in the Zenodo repository, DOI 10.5281/zenodo.7054506 and hyperlink to dataset is 10.5281/zenodo.7054506.

## Abbreviations

TBI: Traumatic brain injury
ICUs: Intensive care units
APACHE: Acute Physiology and Chronic Health Evaluation
SOFA: Sequential organ failure assessment
GCS: Glasgow Coma Scale
OR: Odds ratio
CT: Computed tomography
GSW: Gunshot wound
SW: Stab wound
SAH: Subarachnoid haemorrhage
SDH: Subdural haematoma
EDH: Epidural haematoma
DAI: Diffuse axonal injury
IPH: Intraparenchymal haemorrhage
ICPm: Intra cranial pressure monitoring
EVD: External ventricular derivation
PTE: Pulmonary thromboembolism
ARDS: Acute respiratory distress syndrome
AMI: Acute myocardial infarction
CA: Cardiac arrest
VF: Ventricular fibrillation
SLF: Same-level fall
OLF: Fall from other level
SCI: Spinal cord injure
